# Deciphering Alveolo-Capillary Gas Transfer Disturbances in Patients Recovering from COVID-19 Lung Disease

**DOI:** 10.3390/jpm14070738

**Published:** 2024-07-10

**Authors:** Thông Hua-Huy, Hà Pham-Ngoc, Frédérique Aubourg, Christine Lorut, Nicolas Roche, Anh Tuan Dinh-Xuan

**Affiliations:** 1 Lung Function & Respiratory Physiology Unit, Department of Respiratory Physiology and Sleep Medicine, Assistance Publique—Hôpitaux de Paris, Cochin Hospital, University Paris Cité, 75006 Paris, France; 2 Department of Respiratory Medicine, APHP Centre, Institut Cochin (UMR 1016), Assistance Publique—Hôpitaux de Paris, Cochin Hospital, University Paris Cité, 75006 Paris, France

**Keywords:** COVID-19, pulmonary diffusion, pulmonary function, lung capillary volume, nitric oxide

## Abstract

Impaired lung gas exchange is commonly seen in patients with pulmonary involvement related to SARS-CoV-2 acute infection or post-acute COVID-19 syndrome (PACS). The primary aim of our study was to assess lung gas transfer, measuring the pulmonary diffusion capacity for nitric oxide (D_LNO_) and carbon monoxide (D_LCO_) in all COVID-19 patients. Our secondary aim was to decipher the respective roles of perturbed lung membrane conductance (D_M_) and reduced pulmonary capillary volume (V_C_) in patients with impaired lung gas exchange. From May to October 2020, we measured D_LNO_-D_LCO_ in 118 patients during their post-COVID-19 period (4.6 months after infection) to decipher alveolo-capillary gas transfer disturbances. D_LNO_-D_LCO_ measurement was also performed in 28 healthy non-smokers as controls. Patients were classified into three groups according to the severity (mild, moderate, and severe) of acute COVID-19 infection. Patients with mild COVID-19 had normal lung volumes and airways expiratory flows but impaired pulmonary gas exchange, as shown by the significant decreases in D_LNO_, D_LCO_, D_M_, and V_C_ as compared with controls. V_C_ was significantly impaired and the D_LNO_/D_LCO_ ratio was increased in patients with moderate (n = 4, 11%) and severe COVID-19 (n = 23, 49%). Abnormal membrane conductance was also seen in all three groups of post-COVID-19 patients. These findings suggest a persistent alveolo-capillary gas transfer defect, implying not only reduced membrane conductance but also abnormal pulmonary vascular capacitance in all PACS patients, even those with a milder form of COVID-19 infection.

## 1. Introduction

Between 29 January 2020 and 13 April 2024, more than 704 million people were infected by SARS-CoV-2, which causes coronavirus disease 2019 (COVID-19), and there were over 7 million related deaths worldwide (https://www.worldometers.info/coronavirus/, accessed on 8 July 2024). COVID-19 is usually benign with self-limited upper airway symptoms, but a proportion of patients can develop pneumonia with or without pulmonary embolism, eventually leading to acute hypoxemic respiratory distress syndrome and death. Numerous patients recovering from COVID-19 had long-lasting symptoms such as breathlessness, chest pain, chronic cough, fatigue, and anxiety, known as post-acute COVID-19 syndrome (PACS). Patients with PACS might have persistent lung function abnormalities including lung volume reduction and impaired lung gas exchanges [1,2,3].

Lung gas exchange is usually assessed by measuring the single-breath diffusion capacity of the lungs for carbon monoxide with a standard 10 s breath-hold time (DLCO_10s_) [4]. Many studies found decreased DLCO_10s_ not only during the acute phase of COVID-19 but also up to 6 and 12 months after initial infection [2,5,6,7]. As DLCO_10s_ is a product of the accessible alveolar volume (V_A_) and the carbon monoxide transfer coefficient (KCO), which indicates the efficiency of CO transfer across the alveolar membrane, impaired DLCO_10s_ might result from either reduced V_A_ or low KCO, or both. Reduced V_A_ is associated with a loss of functional alveolar units during the early phase of pneumonia-related condensation, whereas low KCO might be a consequence of interstitial alveolar alteration, vascular injury, or both. Although convenient and reliable, with reproducible results, standard DLCO_10s_ cannot distinguish the parts played by vascular versus interstitial impairment in a given patient with abnormal lung gas exchange [8].

Unlike DLCO_10s_, the single-breath lung diffusion capacity for nitric oxide and carbon monoxide (D_LNO_-D_LCO_) can decipher the respective roles played by alveolar membrane gas conductance (D_M_) and pulmonary capillary volume (V_C_) in lung gas exchange impairment. As a result, D_LNO_-D_LCO_ measurement is useful in systemic sclerosis, precapillary pulmonary hypertension, sarcoidosis, and cystic fibrosis, where alveolar membrane and pulmonary capillary volume differentially affect lung gas exchange. Due to its very high affinity and reactance rate to haemoglobin (ƟNO), NO transfer is more sensitive to alveolar membrane damage, whereas CO transfer is more sensitive to vascular injury [9,10]. Studies investigating D_LNO_-D_LCO_ in PACS patients gave inconsistent results, probably due to differences in the acute COVID-19 severity, delay from COVID-19 to post-infection assessment, and residual symptoms associated with PACS [11,12,13,14,15,16,17,18]. Patients recovering from COVID-19 presented with a greater reduction in membrane gas conductance (D_M_) than in pulmonary capillary volume (V_C_) in some studies [11,12,13,14] whereas other reports found more patients with reduced V_C_ [15,16] or both D_M_ and V_C_ diminutions [17,18].

The aim of our study was two-fold. First, we wanted to decipher the respective roles of perturbed lung membrane conductance, and reduced pulmonary capillary volume in the overall impairment of lung gas exchange seen in patients recovering from COVID-19 infection during the first wave of the pandemic. Secondly, we wanted to see whether impairments in these two parameters differently affect patients with mild, moderate, and severe COVID-19 lung disease.

## 2. Population and Methods

### 2.1. Population

We prospectively enrolled patients recovering from SARS-CoV-2 infection who were seen in the Department of Physiology, Cochin Hospital, Paris, France from 2 May 2020 to 31 October 2020 for follow-up. The date of first symptoms of acute COVID-19 infection ranged from 12 February to 13 May 2020. All patients performed spirometry, body plethysmography, exhaled nitric oxide, and pulmonary diffusion capacity for nitric oxide and carbon monoxide (D_LNO_-D_LCO_) tests on the same day. D_LNO_-D_LCO_ measurement was also performed in 28 healthy, non-smoking subjects serving as controls. Some data from this cohort of patients have been previously published [19,20]. Exclusion criteria were recent respiratory infection (acute bronchitis, bacterial pneumonia less than 4 weeks ago) and medical history of pre-existing diseases that might modify the DLNO/DLCO results, such as interstitial lung disease (ILD) or pulmonary arterial hypertension [10,21]. All pulmonary functional tests (PFT) including D_LNO_-D_LCO_ measurement have been routinely performed in our patients with informed consent. The study complied with our institutional rules, and the need for specific consent, as judged by our local ethics committee was therefore waived [19,20].

Acute SARS-CoV-2 infection was confirmed by multiplex real-time reverse transcription (RT-PCR) on nasopharyngeal swabs, or retrospectively by serological quantification of anti-SARS-CoV-2 IgG antibodies, as noted in patients’ medical files. SARS-CoV-2 infection was classified as mild, moderate, and severe disease, as previously described [20]. Briefly, patients in the mild (disease) group had no sign of pneumonia and no oxygen desaturation. In the moderate group, patients had pneumonia visualized on thoracic scanner (HRCT) with oxygen administration up to 15 L/min. Patients in the severe group were hospitalized in the intensive care unit (ICU) and had received high-flow nasal cannula therapy and/or mechanical ventilation for more than 24 h [20].

### 2.2. Pulmonary Function Test

PFT was routinely performed according to the ATS/ERS recommendations [21], and the Global Lung Function Initiative (GLI) references were used to determine predictive values for spirometry [22], lung volumes [23], and the diffusing capacity of the lungs for carbon monoxide [4]. Obstructive ventilatory defect was defined as a reduced FEV1/FVC ratio (<the 5th percentile of the predicted value), and restrictive ventilatory defect as a TLC reduced below the 5th percentile of the predicted value [24].

### 2.3. Exhaled Nitric Oxide Measurement

All patients performed exhaled NO measurement prior to the pulmonary function test (PFT), as recommended by the American Thoracic Society/European Respiratory Society (ATS/ERS) (ATS/ERS 2005). Fractional exhaled nitric oxide (FeNO) was obtained by different stable expiratory flow rates of 50 mL/s, 100 mL/s, and 150 mL/s (FeNO+ Hyp’air, MGC Diagnostics, Dinant, Belgium). Alveolar concentration of exhaled nitric oxide (CaNO) and maximal conducting airway flux of NO (J’awNO) were calculated using the two-compartment model, as previously detailed [25].

### 2.4. Combined Carbon Monoxide and Nitric Oxide Lung Diffusion (D_LNO_-D_LCO_) Measurement

Single-breath D_LNO_-D_LCO_ measurement (Hyp’air, MGC Diagnostics, Dinant, Belgium) was performed according to recommendations from ATS/ERS [26]. Patients were asked to perform a slow vital capacity (SVC) prior to D_LNO_-D_LCO_ measurement for the quality control of inspired volume (≥90% of SVC). D_LNO_-D_LCO_ measurement was performed using the single-breath procedure in the sitting position with a breath-hold time of 4 s. The concentration of nitric oxide in the inspiration bag was set at 45 ppm (parts per million) to obtain the real value from 40 ppm to 60 ppm, as recommended. Predicted values were calculated according to ATS/ERS guidelines with infinite ƟNO [26,27].

### 2.5. Statistical Analyses

Results are presented as mean and standard deviation, or number [percentage], as appropriate. Statistical analyses were performed using one-way ANOVA with post hoc Tukey test for continuous variables and chi-squared tests for categorical ones. Linear correlations between lung function variables (FVC, FEV1, TLC) and exhaled nitric oxide (CaNO, FeNO, and J’awNO) and the double diffusion D_LNO_-D_LCO_ (including V_A_, T_LNO_, T_LCO_, K_NO_, K_CO_, D_M_, and V_C_) were performed using Pearson’s method. A *p*-value less than 0.05 was considered as statistically significant. All analyses were performed with IBM SPSS Statistics 20.0 (Chicago, IL, USA).

## 3. Results

### 3.1. Studied Population

Between 2 May 2020 and 31 October 2020, 132 patients recovering from COVID-19 infection were enrolled. Twelve patients were excluded since they were not able to perform D_LNO_-D_LCO_ measurements (inspired volume < 90% of slow vital capacity and/or leaky air volume during apnoea). Two patients with systemic sclerosis-associated pulmonary fibrosis were removed from the cohort. Finally, 118 patients (72 men, age 56 ± 13 years) were included in the final analyses. They were classified into three groups according to the severity of initial SARS-CoV-2 infection, as previously described (Table 1).

The severe group had higher percentages of men (78.7%), arterial hypertension (31.9%), and pulmonary embolism during COVID-19 (19.1%) as compared with the mild and moderate groups (*p* < 0.05). There were no significant differences in body mass index, obesity rate, tobacco smoking status, and the delay from COVID-19 to PFT performance (*p* > 0.05). The mean (±standard derivation) time from COVID-19 to a clinical visit with the PFT was 138 days (±37 days).

### 3.2. Pulmonary Function Tests (PFTs) and Exhaled Nitric Oxide (NO) Measurement

The PFT results showed that patients recovering from severe SARS-CoV-2 infection had more risk of a restrictive defect (43%) than patients with moderate (31%) and mild (3%) forms of COVID-19 (*p* < 0.001) (Table 2).

There was also a significant decrease in DLCO_10s_ in the severe group (69% ± 17% of the predicted value) as compared with the moderate and mild groups (79% ± 16% and 89% ± 14% of the predicted value, respectively, *p* < 0.001). As expected, there were more patients with decreased DLCO_10s_ (<lower limit of normal) in the severe group (57.4%) as compared with the moderate (54.3%) and mild groups (13.9%). Overall, 51 patients (43%) exhibited impaired gas transfer 4.6 months after COVID-19 (Table 2).

Exhaled NO measurement was performed in the whole population using the two-compartment method to determine the alveolar concentration of nitric oxide (CaNO) and the bronchial maximal flux of NO (J’awNO). There was no significant difference in any of the exhaled NO parameters (*p* > 0.05) among the three groups of COVID-19 patients (Table 2).

### 3.3. Lung Diffusing Capacities for Nitric Oxide (D_LNO_) and for Carbon Monoxide (D_LCO_) Measurement

We measured the D_LNO_-D_LCO_ in 28 healthy, non-smoker subjects (18 men; age: 50 ± 17 years) serving as the control group. All healthy subjects are members of our departments who volunteered to take part in the study after giving informed consent, as per our institution’s regulatory procedures for PFT measurement. None of them are smokers, nor were they infected by SARS-CoV-2 or affected by any acute or chronic health conditions. Comparing the D_LNO_-D_LCO_ results between the control group and the mild COVID-19 group, we found no difference in alveolar volume (*p* = 0.99), but there was a significant decrease in D_LNO_ (*p* < 0.001), D_LCO_ (*p* < 0.001), K_NO_ (*p* = 0.014), and K_CO_ (*p* = 0.004) in the mild COVID-19 patients. In addition, the D_LNO_/D_LCO_ ratio was significantly higher in the mild COVID-19 group than in the controls (5.57 ± 0.54 versus 5.14 ± 0.31; *p* < 0.001) (Table 3). Similarly, D_M_ and V_C_ were significantly decreased in the mild group as compared with the control group (*p* < 0.001). There were 16 patients (44.4%) with reduced D_M_ and 2 patients (5.6%) with reduced V_C_ among the patients with mild COVID-19 (Table 3).

Table 4 presents a comparison of all the D_LNO_-D_LCO_ parameters among the three groups of patients. Accessible alveolar volume (V_A_) decreased with the severity of COVID-19, as did D_LNO_, D_LCO_, K_NO_, K_CO_, D_MCO_, and Vc. However, there was no significant difference in the D_LNO_/D_LCO_ ratio among the three groups of patients (*p* = 0.513). In the severe group, there were 23 patients (48.9%) with decreased V_C_ and 44 patients (93.6%) with decreased D_M_. Globally, there were 83 patients with decreased D_M_ (70%) and 29 patients with reduced V_C_ (25%) (Table 4).

As expected, we found good correlations between variables from the D_LNO_-D_LCO_ measurement and those from the pulmonary function test in patients recovering from SARS-CoV-2 infection, with correlation coefficients varying from 0.359 to 0.868 (Table 5).

## 4. Discussion

The impairment of pulmonary gas exchange in COVID-19 patients 3 to 6 months after acute infection with SARS-CoV2 observed in our study is consistent with previously published reports [1]. The originality of our data lies in the ability to revisit the mechanisms of this impairment at the level of the alveolo-capillary membrane, highlighting the involvement of the pulmonary microvasculature even in patients with mild COVID-19 related lung disease. In all three groups of patients with different levels of acute COVID-19 severity, D_LNO_-D_LCO_ was able to decipher the respective roles of pulmonary vascular injury (V_C_), interstitial lung damage (D_M_), and accessible alveolar gas exchange volume (V_A_). First, COVID-19 patients with mild disease maintained a normal alveolar volume yet had impaired lung gas exchange, as shown by the mild reduction in D_LNO_ and marked reduction in D_LCO_. Both D_M_ and V_C_ were markedly reduced in patients with mild disease as compared with the controls (Table 3). Secondly, COVID-19 patients with moderate disease had a mild reduction in V_A_, and worsened D_LNO_ and D_LCO_ impairment as compared with patients with mild disease. Interestingly, severe impairment of Vc was already detected in patients with moderate disease, contrasting with less severe alteration of D_M_. Finally, COVID-19 patients with severe disease had a marked reduction in alveolar volume and severe impairments in all lung gas transfer parameters (D_LNO_, D_LCO_, D_M_, and V_C_) (Table 4).

During the early phase of the pandemic, several COVID-19 patients presented with acute respiratory distress syndrome (ARDS) with severe hypoxemia as a consequence of multiple pathophysiological processes, as shown by autopsy findings [28]. Mechanisms of pulmonary shunt include disordered architecture of the pulmonary capillary beds, characterized by intussusceptive angiogenesis and the presence of multiple pulmonary micro-thrombotic lesions. These vascular injuries can induce an impaired pulmonary capillary volume (V_C_). Persistent alveolar inflammation with subsequent abnormal lung extracellular matrix remodelling and interstitial fibrotic damage might reduce the alveolo-capillary membrane conductance (D_M_) in post-COVID-19 patients [29]. Previous studies using D_LNO_-D_LCO_ measurement have to date yielded contrasting results regarding membrane and/or vascular defects [11,12,13,14,15,16,17,18].

In a study [11] of 94 patients assessed at 8 months after hospital discharge for COVID-19 pneumonia, 57% had a decrease in D_LNO_ while only 20% had an impairment in the standard DLCO measurement (DLCO_10s_ in our study). In-depth analyses suggested a loss of functional alveolar units along with alveolar membrane damage as the predominant impaired gas exchange mechanism, while the lung capillary volume remained relatively preserved [11,14]. These findings were consistent with those of Nunez-Fernandez et al. [12], who reported that combined D_LNO_-D_LCO_ measurement was more sensitive than DLCO alone in detecting a gas exchange abnormality at 3 months after discharge. In support of this conclusion, the authors found that DLCO was within normal limits in 40% of the patients with a decreased D_LNO_. In our study, 43% of the post-COVID-19 patients (n = 51) had a decreased DLCO_10s_ whereas only 37% of them (n = 44) had a reduced D_LNO_, suggesting that microvascular damage might be the principal mechanism of pulmonary gas exchange perturbances. Likewise, another study looking at 33 patients recovering from severe COVID-19 also found a prevailing vascular abnormality, namely gas exchange impairment [16], where 76% of the patients had a high D_LNO_/D_LCO_ ratio (>110% of the theoretical value) [10] and 73% had a reduced V_C_ (<lower limit of normal). This finding was in line with our results, as evidenced by the high proportion of patients with impaired D_LCO_ (85%) and decreased V_C_ (49%) in the severe group as compared with the mild (17% and 6%, respectively) and moderate groups (46% and 11%, respectively) (Table 4).

A real-life study of 204 patients with post-COVID-19 syndrome (PACS), using the cardiopulmonary exercise test (CPET) and D_LNO_-D_LCO_ measurement at about 6 months (171 ± 85 days) after the end of the acute COVID-19 infection, has shown that inefficiency of ventilation during exercise was associated with reduced V_C,_ suggesting endothelial vascular dysfunction as the predominant cause of gas exchange impairment in PACS [30]. Similarly, abnormal gas exchange with vascular dysfunction, characterized by decreased V_C_ and a high D_LNO_/D_LCO_ ratio, was found to be related to long-lasting dyspnoea in patients recovering from COVID-19 [15]. Other reports demonstrated that D_M_ and D_LNO_ were more reduced in patients with dyspnoea at 12 months after healing from COVID-19 [13,14]. One recent study measuring single-breath D_LNO_-D_LCO_ in post-COVID-19 patients at 6 and 12 months after acute SARS-CoV-2 infection showed that 60% of these patients had at least one abnormal D_LNO_ or D_LCO_ value, but there was no consistent pattern of contribution of lung membrane conductance D_M_ or pulmonary capillary volume V_C_ to the underlying mechanism [18]. Seccombe et al. also reported a decrease in both D_M_ and V_C_ 2 months after an acute severe COVID-19 infection. However, abnormal alveolar membrane conductance (Dm) seemed to improve significantly at 4 months and 8 months whereas reduced lung capillary volume (Vc) persisted [17]. Altogether, abnormal gas exchange appears to persist in PACS patients with an initial COVID-19 infection of any severity, but there is no fixed pattern regarding interstitial and/or vascular damage. Single-breath D_LNO_-D_LCO_ measurement represents an interesting technique to differentiate between lung membrane conductance alteration and pulmonary vascular volume anomaly as causes of impairment in pulmonary gas exchange.

Our study had some limitations. We did not study the correlation between D_LNO_-D_LCO_ parameters and lung scan findings. Other researchers found a good inverse correlation between D_LNO_ and D_LCO_, with persistent CT ground glass opacities and mean lung attenuation [11]. The authors of another study [15] excluding all patients with persistent CT abnormalities still found a significant decrease in D_LNO_, D_LCO_, and V_C_ in patients with residual dyspnoea (as compared with non-dyspnoeic patients), showing that impaired gas exchange can persist even in patients with seemingly normal lung CT images [15].

## 5. Conclusions

Our study confirmed that lung gas transfer impairment is present in all COVID-19 patients, even in the mildest form of the disease when the alveolar volume is still normal. The pulmonary capillary volume (V_C_) is the most affected parameter, with severe impairment starting in patients with moderately (but not severely) impaired lung volumes. An alteration of V_C_, as an early and sensitive marker of altered lung gas exchange, suggests a pivotal role of vascular abnormality in COVID-19 and post-COVID-19 lung disease. We hypothesize that early detection and treatment of pulmonary vascular injuries may (1) slow down or even halt the progression of COVID-19 pneumonia in its mild-to–severe forms and (2) decrease respiratory post-COVID-19 manifestations.

## Figures and Tables

**Table 1:** Demographics and comorbidities of patients recovering from SARS-CoV-2 infection.

	Mild	Moderate	Severe	*p*-Value
	n = 36	n = 35	n = 47	(ANOVA)
Age; years	51 ± 14	60 ± 12 *	57 ± 11	0.015
Male; n (%)	12 (33.3)	23 (65.7)	37 (78.7)	<0.001
Height; cm	167 ± 9	171 ± 9	170 ± 8	0.127
Weight; kg	74 ± 18	82 ± 14	77 ± 12	0.084
BMI; kg.m^−2^	26.3 ± 5	27.8 ± 4.3	26.7 ± 4.2	0.358
Obesity; n (%)	8 (22.2)	11 (31.4)	8 (17)	0.305
Tobacco				
Non-smoker; n (%)	28 (77.8)	25 (71.4)	36 (76.6)	
Active; n (%)	4 (11.1)	3 (8.6)	2 (4.3)	0.649
Former; n (%)	4 (11.1)	7 (20)	9 (19.1)	
Time from COVID-19; days	140 ± 38	143 ± 42	133 ± 32	0.456
Co-morbidities				
Lung disease; n (%)	9 (25)	8 (22.9)	6 (12.8)	0.32
Asthma; n (%)	8 (22.2)	4 (11.4)	0 (0)	0.004
COPD; n (%)	0 (0)	1 (2.9)	1 (2.1)	0.62
Sleep apnoea syndrome; n (%)	2 (5.6)	3 (8.6)	2 (4.3)	0.71
Heart disease; n (%)	0 (0)	2 (5.7)	6 (12.8)	0.07
Arterial hypertension; n (%)	3 (8.3)	9 (25.7)	15 (31.9)	0.04
Diabetes; n (%)	3 (8.3)	8 (22.9)	10 (21.3)	0.2
Pulmonary embolism during COVID-19	0 (0)	4 (11.4)	9 (19.1)	0.004

Results are presented as mean ± standard derivation, or number (percentage), as appropriate. Comparisons among three groups of COVID-19 patients with mild, moderate, and severe disease were performed using one-way ANOVA with post hoc Tukey test for continuous variables and chi-squared test for categorical ones. BMI: body mass index; COPD: chronic obstructive pulmonary disease; n: number (of patients); %: percentage of patients relative to total number of patients in the corresponding group (mild, moderate, or severe). *: *p* < 0.05 (Tukey test versus mild COVID-19 group).

**Table 2:** Pulmonary function test (PFT) and exhaled nitric oxide (NO) measurement in patients recovering from SARS-CoV-2 infection.

	Mild	Moderate	Severe	*p*-Value
	n = 36	n = 35	n = 47	(ANOVA)
Body plethysmography				
TLC; % pred	99 ± 12	90 ± 16 **^§^	81 ± 12 ***	<0.001
FRC; % pred	103 ± 20	95 ± 21	93 ± 19	0.07
RV; % pred	125 ± 24	106 ± 17 **	96 ± 24 ***	<0.001
Restrictive pattern TLC < LLN-GLI; n (%)	1 (2.8)	11 (31.4)	20 (42.6)	<0.001
Spirometry				
FVC; % pred	94 ± 14	87 ± 18	82 ± 14 **	0.002
FEV1; % pred	93 ± 12	88 ± 18	87 ± 15	0.18
FEV1/FVC; %	80 ± 7	79 ± 7 ^§§^	84 ± 7 **	0.001
Obstructive pattern FEV1/FVC < LLN-GLI; n (%)	1 (2.8)	1 (2.9)	1 (2.1)	0.97
Single breath DLCO_10s_				
DLCO_10s_; %pred	89 ± 14	79 ± 16 *^§^	69 ± 17 ***	<0.001
KCO_10s_; % pred	93 ± 10	93 ± 14	86 ± 17	0.04
V_A10s_; % pred	95 ± 12	85 ± 17 **	80 ± 13 ***	<0.001
Lung diffusion impairment DLCO_10s_ < LLN-GLI; n (%)	5 (13.9)	19 (54.3)	27 (57.4)	<0.001
Exhaled nitric oxide (£)	n = 33	n = 35	n = 46	
CaNO (ppb)	4.15 ± 1.71	4.56 ± 2.81	4.72 ± 2.02	0.53
FeNO (ppb)	21.2 ± 8.8	25.2 ± 13.9	21.8 ± 8.7	0.23
J’awNO (nL/mn)	52.3 ± 24.3	65.9 ± 41.5	53.8 ± 25.8	0.13

Results are presented as mean ± standard derivation, or number (percentage), as appropriate. Comparisons between the three groups of COVID-19 patients used one-way ANOVA with post hoc Tukey test for continuous variables and chi-squared test for categorical ones. NO: nitric oxide; BMI: body mass index; PFT: pulmonary function test; TLC: total lung capacity; FRC: functional residual capacity; RV: residual volume; LLN: lower limit of normal; GLI: global lung function initiative; FVC: forced vital capacity; FEV_1_: forced expiratory volume in one second; DLCO_10s_: lung diffusion capacity for carbon monoxide; KCO_10s_: coefficient of lung diffusion for carbon monoxide; V_A10s_: alveolar volume; % pred: percentage of predictive values. CaNO: alveolar concentration of exhaled nitric oxide; FeNO: fractional expired nitric oxide concentration at expiratory flow rate of 50 mL/s; J’awNO: maximal conducting airway flux of NO (FeNO+ Hyp’air, MGC Diagnostics, Belgium). (£) Three patients in mild group and one patient in severe group did not satisfy the quality requirements for CaNO measurement. *: *p* < 0.05; **: *p* < 0.01; ***: *p* < 0.001 (Tukey test versus mild COVID-19 group); **^§^**: *p* < 0.05; **^§§^***: p* < 0.01 (Tukey test versus severe COVID-19 group).

**Table 3:** D_LNO_-D_LCO_ results in healthy controls and patients recovering from mild COVID-19 infection.

Single-Breath NO Uptake Infinite ƟNO	Healthy	Mild	*p*-Value
	n = 28	n = 36	
V_A_; litre	5.6 ± 1.1	5.6 ± 1.0	0.88
V_A_; % pred	98 ± 9	98 ± 12	0.99
V_A_ < LLN; n (%)	0	1 (2.8)	N/A
D_LNO_; mL.min^−1^.mmHg^−1^	131 ± 38	119 ± 34	0.18
D_LNO_; % pred	106 ± 14	93 ± 16	0.001
D_LNO_ < LLN; n (%)	0	3 (8.3)	N/A
D_LCO_; mL.min^−1^.mmHg^−1^	25.6 ± 7.9	21.6 ± 6.2	0.023
D_LCO_; % pred	100 ± 15	82 ± 14	<0.001
D_LCO_ < LLN; n (%)	0	6 (16.7)	N/A
D_LNO_/D_LCO_	5.14 ± 0.31	5.57 ± 0.54	<0.001
D_LNO_/D_LCO_ > ULN (5.75)	0	10 (27.8)	0.003
K_NO_; mL.min^−1^.mmHg^−1^.L^−1^	23.3 ± 4	20.9 ± 3.4	0.014
K_NO_; % pred	103 ± 14	93 ± 12	0.014
K_NO_ < LLN; n (%)	0	0 (0)	N/A
K_CO_; mL.min^−1^.mmHg^−1^.L^−1^	4.5 ± 0.85	3.8 ± 0.7	<0.001
K_CO_; % pred	96 ± 15	80 ± 12	0.001
K_CO_ < LLN; n (%)	0	11 (30.6)	N/A
D_M_; mL.min^−1^.mmHg^−1^	66.7 ± 19.8	60.5 ± 17.2	0.18
D_M_; % pred	62.3 ± 8.6	52.3 ± 9.5	<0.001
D_MCO_ < LLN; n (%)	0	16 (44.4)	N/A
V_C_; mL	79.7 ± 23.8	63.7 ± 16.9	0.003
V_C_; % pred	112.5 ± 22.1	91.3 ± 18	<0.001
V_C_ < LLN; n (%)	0	2 (5.6)	N/A

Results are presented as mean ± standard derivation, or number (percentage), as appropriate. ƟNO: reaction rate of nitric oxide to haemoglobin; V_A_: alveolar volume; D_LNO_: transfer factor for nitric oxide; D_LCO_: transfer factor for carbon monoxide; K_NO_: nitric oxide transfer coefficient; K_CO_: carbon monoxide transfer coefficient by double diffusion (4 s apnoea); D_M_: membrane conductance for carbon monoxide; V_C_: pulmonary capillary (blood) volume; LLN: lower limit of normal; GLI: global lung function initiative. N/A: not applicable.

**Table 4:** D_LNO_-D_LCO_ results of three groups of patients who recovered from SARS-CoV-2 infection.

Single-Breath NO Uptake Infinite ƟNO	Mild	Moderate	Severe	*p*-Value (ANOVA)
	n = 36	n = 35	n = 47	
V_A_; litre	5.6 ± 1.0	5.5 ± 1.3	5.0 ± 1.0 *	0.027
V_A_; % pred	98 ± 12	87 ± 16 ^§§^	80 ± 13 ***	<0.001
V_A_ < LLN; n (%)	1 (2.8)	10 (28.6)	17 (36.2)	0.001
D_LNO_; mL.min^−1^.mmHg^−1^	119 ± 34	110 ± 34 ^§§^	90 ± 25 ***	<0.001
D_LNO_; % pred	93 ± 16	83 ± 20 *^§§^	63 ± 16 ***	<0.001
D_LNO_ < LLN; n (%)	3 (8.3)	9 (25.7)	32 (68.1)	<0.001
D_LCO_; mL.min^−1^.mmHg^−1^	21.6 ± 6.2	19.9 ± 6.2 ^§§^	16 ± 4.9	<0.001
D_LCO_; % pred	82 ± 14	73 ± 16 *^§§^	56 ± 15 ***	<0.001
D_LCO_ < LLN; n (%)	6 (16.7)	16 (45.7)	40 (85.1)	<0.001
D_LNO_/D_LCO_	5.57 ± 0.54	5.6 ± 0.64	5.71 ± 0.66	0.513
D_LNO_/D_LCO_ > ULN (5.75)	10 (27.8)	10 (28.6)	15 (31.9)	0.9
K_NO_; mL.min^−1^.mmHg^−1^.L^−1^	20.9 ± 3.4	20 ± 3.5 ^§^	17.9 ± 3.5 ***	<0.001
K_NO_; % pred	93 ± 12	94 ± 13 ^§§§^	82 ± 15 **	<0.001
K_NO_ < LLN; n (%)	0 (0)	1 (2.9)	15 (31.9)	<0.001
K_CO_; mL.min^−1^.mmHg^−1^.L^−1^	3.8 ± 0.7	3.6 ± 0.7 ^§^	3.2 ± 0.7 ***	<0.001
K_CO_; % pred	80 ± 12	80 ± 12 ^§§§^	69 ± 14 **	<0.001
K_CO_ < LLN; n (%)	11 (30.6)	7 (20.0)	25 (53.2)	<0.001
D_M_; mL.min^−1^.mmHg^−1^	60.5 ± 17.2	55.9 ± 17.1 ^§^	45.5 ± 12.7 ***	<0.001
D_M_; % pred	52.3 ± 9.5	46.1 ± 13.4 *^§§§^	33.3 ± 9.2 ***	<0.001
D_M_ < LLN; n (%)	16 (44.4)	23 (65.7)	44 (93.6)	<0.001
V_c_; mL	63.7 ± 16.9	56 ± 16.8 ^§^	45.6 ± 14.1 ***	<0.001
V_c_; % pred	91.3 ± 18	79.5 ± 19 *^§§^	65.7 ± 18.8 ***	<0.001
V_c_ < LLN; n (%)	2 (5.6)	4 (11.4)	23 (48.9)	<0.001

Results are presented as mean ± standard derivation, or number (percentage), as appropriate. ƟNO: reaction rate of nitric oxide to haemoglobin; V_A_: alveolar volume; D_LNO_: transfer factor for nitric oxide; D_LCO_: transfer factor for carbon monoxide; K_NO_: nitric oxide transfer coefficient; K_COdd_: carbon monoxide transfer coefficient by double diffusion (NO uptake measurement); D_M_: membrane conductance for carbon monoxide; V_C_: pulmonary capillary (blood) volume; LLN: lower limit of normal; GLI: global lung function initiative. *: *p* < 0.05; **: *p* < 0.01; ***: *p* < 0.001 (Tukey test versus mild COVID-19 group); **^§^**: *p* < 0.05; **^§§^**: *p* < 0.01; **^§§§^**: *p* < 0.001 (Tukey test versus severe COVID-19 group). N/A: not applicable.

**Table 5:** Relationships between variables from D_LNO_-D_LCO_ measurement and those from pulmonary function test in patients recovering from SARS-CoV-2 infection.

	V_A_	D_LNO_	D_LCO_	D_M_	V_C_
TLC	0.868 ***	0.671 ***	0.644 ***	0.661 ***	0.578 ***
FRC	0.685 ***	0.437 **	0.395 **	0.402 **	0.359 **
RV	0.583 ***	0.474 **	0.441 **	0.471 **	0.394 **
FVC	0.786 ***	0.621 ***	0.604 ***	0.582 ***	0.553 ***
FEV_1_	0.701 ***	0.509 ***	0.504 ***	0.456 **	0.486 ***

PFT variables were analysed using percentages of predictive values. Relationship between every two variables was analysed using Pearson’s two-tailed correlation test, and results are presented as rho’s correlation coefficient. **: *p* < 0.01; ***: *p* < 0.001. PFT: pulmonary function test; V_A_: alveolar volume; D_M_: membrane conductance for carbon monoxide; V_C_: pulmonary capillary (blood) volume; TLC: total lung capacity; FRC: functional residual capacity; RV: residual volume; FVC: forced vital capacity; FEV_1_: forced expiratory volume in one second.

## Data Availability

Data were extracted from the patients’ individual records before anonymization. They are stored in the authors’ database, but they are not publicly accessible to ensure patients’ confidentiality is fully protected.
